# Individual Participant Data (IPD) Meta-analyses of Randomised Controlled Trials: Guidance on Their Use

**DOI:** 10.1371/journal.pmed.1001855

**Published:** 2015-07-21

**Authors:** Jayne F. Tierney, Claire Vale, Richard Riley, Catrin Tudur Smith, Lesley Stewart, Mike Clarke, Maroeska Rovers

**Affiliations:** 1 MRC Clinical Trials Unit at University College London, London, United Kingdom; 2 Research Institute of Primary Care and Health Sciences, Keele University, Keele, United Kingdom; 3 MRC North West Hub for Trials Methodology Research, Department of Biostatistics, University of Liverpool, Liverpool, United Kingdom; 4 Centre for Reviews and Dissemination, University of York, York, United Kingdom; 5 Centre for Public Health, Queens University Belfast, Belfast, United Kingdom; 6 Radboud Institute for Health Sciences, Radboud University Medical Center, Nijmegen, the Netherlands

## Abstract

Jayne Tierney and colleagues offer guidance on how to spot a well-designed and well-conducted individual participant data meta-analysis.

**Summary Points**

- Systematic reviews are most commonly based on aggregate data extracted from publications or obtained from trial investigators.
- Systematic reviews involving the central collection and analysis of individual participant data (IPD) usually are larger-scale, international, collaborative projects that can bring about substantial improvements to the quantity and quality of data, give greater scope in the analyses, and provide more detailed and robust results.
- The process of collecting, checking, and analysing IPD is more complex than for aggregate data, and not all IPD meta-analyses are done to the same standard, making it difficult for researchers, clinicians, patients, policy makers, funders, and publishers to judge their quality.
- Following our step-by-step guide will help reviewers and users of IPD meta-analyses to understand them better and recognise those that are well designed and conducted and so help ensure that policy, practice, and research are informed by robust evidence about the effects of interventions.

## Background

Systematic reviews provide an objective and reliable way of summarising research evidence. They collate all the studies relevant to a particular research question, using explicit, transparent, and systematic methods in order to minimise bias, and may or may not include a meta-analysis, to combine the results of these studies. Systematic reviews are most commonly based on aggregate data extracted from publications or obtained from investigators [1]. These aggregate data represent a summary of the individual participant or patient data (IPD) for each study and may include, for example, intervention effect estimates (e.g., odds ratios or hazard ratios) for different outcomes and average patient characteristics (e.g., the mean age of participants or the proportion of women). This limits the analyses that are possible and may also reduce power. Moreover, the availability and quality of such data may vary across studies, and this can affect the reliability of meta-analysis results [2].

Systematic reviews and meta-analyses of IPD have most commonly been large-scale, international, collaborative projects involving the central collection and reanalysis of the original data on each participant, from all of the relevant trials [3–5]. Most have focussed on assessing the efficacy or effectiveness of treatments or other interventions and therefore have been based on randomised controlled trials (RCTs). The IPD approach not only can bring about substantial improvements to the quantity and quality of data—for example, by including more trials, participants, and outcomes—but also enables standardisation of outcomes across trials and detailed data checking (Table 1, [3–5]). IPD also give greater scope and flexibility in the analyses, including, importantly, the ability to investigate whether an intervention is more or less effective for different types of participant (Table 1, [3–5]). These aspects help provide more in-depth exploration and more detailed and robust meta-analysis results, which can differ from those based on aggregate data (e.g., [6–9]). Additionally, collaboration with investigators supplying trial data can lead to more complete identification of relevant trials and a broader interpretation and endorsement of the results (Table 1, [3–5]).

**Table 1:** Advantages of using an IPD rather than aggregate data approach to systematic review and meta-analysis of RCTs.

Aspect of Systematic Review/Meta-analysis	Advantages of the IPD Approach
Trial inclusion	Asking the IPD collaborative group (of trialists and other experts in the clinical field) to supplement the list of identified trials
	Clarify trial eligibility with trial investigators
Data quality	Include trials that are unpublished or not reported in full
	Include unreported data, e.g., more outcomes per trial and more complete information on those outcomes, and data on participants excluded from trials analyses
	Check the integrity of trial IPD and resolve any queries with trial investigators
	Derive standardised outcome definitions across trials or translate different definitions to a common scale
	Derive standardised classifications of participant characteristics or their disease/condition or translate different definitions to a common scale
	Update follow up of time-to-event outcomes beyond that reported
Risk of bias	Clarify trial design, conduct, and analysis methods with trial investigators
	Check risk of bias of trial IPD and obtain extra data when necessary
Analysis	Analyse all important outcomes
	Determine validity of analysis assumptions with IPD, e.g., proportionality of hazards for a Cox model
	Derive measures of effect directly from the IPD
	Use a consistent unit of analysis for each trial
	Apply a consistent method of analysis for each trial
	Conduct more detailed analysis of time-to-event outcomes, e.g., generating Kaplan Meier curves
	Achieve greater power for assessing interactions between effects of interventions and participant or disease/condition characteristics
	Conduct more complex analyses not (usually) possible with aggregate data, e.g., simultaneous assessment of the relationship between multiple trial and/or participant characteristics and effects of interventions
	Use nonstandard models or measures of effect
	Use IPD for secondary clinical questions, e.g., to explore the natural history of disease, prognostic factors, or surrogate outcomes
Interpretation	Discuss the implications for clinical practice and research with a multidisciplinary group of collaborators including trial investigators who supplied the data

Given their considerable advantages, meta-analyses that are based on IPD have been called the “gold standard” of systematic review [10], and their use to assess the effects of interventions from RCTs has increased [11], across a range of health care areas [5] and in both higher- and lower-resource settings. However, the process of collecting, checking, and analysing IPD is more complex than for aggregate data, and recent evidence suggests that not all IPD meta-analyses are done or reported to the same standard [11,12]. Also, previous guidance, mainly aimed at systematic reviewers, has considered only some of the biases that can arise in aggregate data meta-analyses and how the collection of IPD can help resolve such biases and facilitate understanding (e.g., [3–5]). These issues can make it difficult for researchers, clinicians, patients, policy makers, funders, and publishers to judge the quality of IPD meta-analyses. Therefore, it may hinder their conduct, dissemination, uptake in clinical guidelines [13] policy and practice and their influence on trials [14]. This guidance will help a variety of stakeholders understand, appraise, and make best use of IPD meta-analyses that summarise the efficacy of interventions.

## Is It Part of a Systematic Review?

A key component of appraising an IPD review or meta-analysis of efficacy is determining whether it is part of a systematic review [3–5]. Some hallmarks are the following:

- clear research question, qualified by unambiguous eligibility criteria relating to participants, interventions, comparisons, and outcomes
- systematic and comprehensive search strategy, to ensure that all relevant trials are identified
- consistent approach to data collection, including an explanation of which items were requested
- assessment of the quality or risk of bias of included trials

To limit bias, ensure transparency, and allow scrutiny, all such methods should have been prespecified in a protocol, and ideally this would have been registered [15], published, or otherwise made available for inspection.

Early systematic reviews and meta-analyses based on IPD were commonly described as “overviews” or “pooled analyses” (e.g., [16,17]), and subsequently, “IPD meta-analysis” became the preferred term (and is still widely used). However, with the emergence of more ad hoc IPD meta-analyses [12], researchers have begun to utilise “systematic review and meta-analysis of IPD” to flag those in the context of a systematic review (e.g., [18]).

## Were All Eligible Trials Identified?

Clear eligibility criteria are needed to determine why particular trials were included in an IPD meta-analysis and, therefore, whether it gives an unbiased or otherwise representative view of the evidence (Table 2). For example, a review of 31 IPD meta-analyses found that 29% had an unclear or unduly selective approach to including studies [12]. Of course, there may be situations in which it is reasonable to exclude relevant trials. For example, for cardiovascular disease, in which trials are often very large, it may make sense to exclude small trials that would contribute very little evidence and take considerable effort to obtain. Importantly, the intention to exclude trials on this sort of basis should form part of the prospective eligibility criteria.

**Table 2:** Biases that can affect systematic reviews and meta-analyses of IPD and aggregate data (AD), and steps taken to investigate and/or minimise these.

Type of Bias	Definition	Steps That Are Taken to Investigate and Minimise Bias			
			Usual with both AD and IPD approaches	Usual with IPD approach but may be possible with AD approach *	Only with IPD approach
Study selection bias	Systematic differences between results of trials that are and are not selected for inclusion	Prospectively define eligibility criteria	✓		
		Clarify eligibility with trial protocol or trialist		✓	
Publication bias	Systematic differences between results of trials that are and are not published	Include all eligible trials irrespective of publication status		✓	
Data availability bias	Systematic difference between the results of trials for which data were and were not available	Include data for all eligible trials		✓	
		Investigate/discuss the impact of trials for which data were not available		✓	
Participant selection bias	Systematic differences between comparison groups in participant characteristics that can lead to differences in prognosis and/or responsiveness to treatment(Prevented by random allocation and allocation concealment)	Clarify the randomisation methods, i.e., sequence generation and allocation concealment with trial protocol or trialist		✓	
		Exclude “nonrandomised” trials		✓	
		Check for unusual allocation patterns or distributions of participant characteristics			✓
		Exclude trials with inappropriate allocation			✓
		Exclude nonrandomised participants from trial IPD			✓
Performance and detection bias	Systematic differences between comparison groups in the care received or provided or in how outcomes are ascertained (Prevented by blinding study participants, care givers, and outcome assessors to the allocated treatment. Note this is not possible for all interventions, e.g., surgery, and is less important for objective outcomes, e.g., mortality)	Obtain more complete information on blinding and outcome assessment from trialist and/or protocol		✓	
Attrition bias	Systematic differences between comparison groups in the dropout or exclusion of participants (Prevented by the maintenance of all participants in the trial and trial analysis)	Include data on all randomised participants, irrespective of whether they were included in trial analyses		✓	
		Analyse all trials according to the allocated intervention (“intention to treat”)		✓	
		Check for “missing” participants and unusual patterns of dropout or exclusion			✓
		Prespecify any reasonable participant exclusions and apply consistently across trials			✓
Outcome reporting or availability bias	Systematic differences between results of reported/available and unreported/unavailable outcomes(Prevented by making results for all study outcomes available)	Check which outcomes were collected in a trial with protocol and/or trialist		✓	
		Include data for all relevant outcomes		✓	

* Only possible with AD reviews if detailed, high-quality data and/or other information can be obtained from trial protocols, trial reports or directly from trial investigators.

Given the substantial evidence that publication of research studies, including RCTs, is influenced by the nature of study results [19], all relevant published and unpublished trials should be sought to avoid bias (Table 2). In 11 IPD meta-analyses in cancer, 45 of the 120 included RCTs were either unpublished or published in non-English language journals, book chapters, or conference abstracts [20]; the so-called “grey literature.” Not including these trials would have produced meta-analysis results that were more in favour of the research treatments than results based on all available trials [20]. Most differences were modest, but in one meta-analysis, there was stronger evidence that postoperative radiotherapy was detrimental to the survival of patients with non-small cell lung cancer when all trials (hazard ratio = 1.21, 95% CI = 1.05–1.39, *p* = 0.001) rather than only fully published trials were included (hazard ratio = 1.13, 95% CI = 0.95–1.34, *p* = 0.066). Yet, IPD meta-analyses do not necessarily include trials published in the grey literature [12], and in the absence of an explicit search strategy (e.g., [21]), it can be difficult to judge whether all trials were identified. Thus, IPD meta-analyses with inappropriately restrictive or absent eligibility criteria and/or search strategies that do not seek all relevant trials need to be viewed with caution.

Direct contact with trial investigators, as part of the IPD approach, can help identify trials that may not be easily identifiable by other forms of searching [3,4] and clarify the eligibility of individual trials (Table 1, [3–5]). It should be noted, however, that even with researchers’ best efforts, all trials may not come to light, and the potential for bias remains. For example, in a systematic review and meta-analysis in which no unpublished trials were identified [22], funnel plot asymmetry suggested that some small studies might be missing [12].

## Were IPD Obtained from Most Trials?

It may be difficult to obtain IPD for all trials identified, because trial investigators do not respond, do not have access to the IPD, or refuse to participate. If nonparticipation is born of a desire to bury unfavourable results, not including such trials could bias a meta-analysis, whereas if it is to avoid providing trials of poor quality, then not including these trials might make a meta-analysis more robust. Aiming to obtain a large proportion of the eligible trials and participants will both counter bias and enable exploration of any quality issues and so will likely provide the most reliable and precise assessment of the effects of an intervention. Upwards of 90% of eligible participants has been suggested as a suitable target to achieve [3], but 33% of a sample of IPD meta-analyses included less than 80% of the data requested [12]. Of course, if the available data can be shown to provide sufficient power to detect an effect reliably, then a lower threshold may be acceptable. With substantial data missing, there is no guarantee that the meta-analysis results will be representative of those based on all data, making data availability bias a possibility. This should be investigated (Table 2), preferably by comparing or combining results based on IPD with those based on aggregate data, to check for coherence and consistency [23]. For example, an IPD meta-analysis in non-Hodgkin lymphoma suggested no clear improvement in survival with high-dose compared to conventional-dose chemotherapy (hazard ratio = 1.14, 95% CI = 0.98–1.34, *p* = 0.401) [24]. Results based on aggregate data were obtained for four of the five trials with missing IPD, and adding these gave greater confidence in the conclusion that high-dose chemotherapy does not affect survival (hazard ratio = 1.05, 95% CI = 0.92–1.19, *p* = 0.157) [24]. Funnel plots may also help indicate the potential impact of both known and unknown missing trials on the results [25] but would not be appropriate in a meta-analysis including less than ten trials [25] or few trials with statistically significant results or one in which heterogeneity has been observed [26]. As a bare minimum, the proportion of trials and participants for which IPD are available and the reasons for unavailability of data should be a standard part of IPD meta-analysis reporting [27].

## Was the Integrity of the IPD Checked?

While there is little opportunity to check the accuracy of aggregate data, IPD can be checked for missing, invalid, out-of-range, or inconsistent items and for discrepancies with any trial publication (Table 1, [3–5]). This would highlight, for example, a participant having an unusually old age or a date of randomisation prior to the date the trial started or instances in which sequentially numbered participants were absent from a dataset. Although similar checks will have been applied during trial conduct, occasionally anomalies still arise. Ideally, these should be queried and, where possible, resolved with the trial's personnel, to improve data quality and ensure that trials are represented accurately.

## Were the Analyses Prespecified in Detail?

As IPD meta-analyses offer the potential for substantially greater numbers of analyses, there is a greater risk that data might be interrogated repeatedly until the desired results are obtained, these being the results that are published. Therefore, it is important that the detailed methods of analysis are prespecified in a protocol or analysis plan and are clearly reported upon publication, including the following:

- primary and secondary outcomes and their definitions
- methods for analysis of efficacy/effectiveness, including those for exploring the impact of trial or participant characteristics
- methods for quantifying and accounting for heterogeneity
- methods for checking IPD and assessing the risk of bias of trials

This is not to say that unplanned analyses are unjustified or invalid; rather, they can play an important role in explaining or adding to per-protocol results. However, such exploratory analyses do need to be justified and clearly labelled as such in any report of the results.

## Was the Risk of Bias of Included Trials Assessed?

Checking the quality of included trials is now a key component of systematic reviews, so that any issues found can be either rectified or taken into account when interpreting the results, and well-conducted IPD meta-analyses are no exception. For aggregate data reviews, this is frequently achieved using the risk of bias tool developed by Cochrane [28,29], based on information provided in trial reports. Seeking additional information from trialists can improve risk of bias assessments associated with, for example, the randomisation procedures or the completeness of outcome data [30,31]. Requesting such clarification tends to be more a feature of IPD rather than aggregate data reviews. Furthermore, checking the IPD directly can provide more reliable investigations of key potential biases, some of which might be reduced or alleviated in the process (Table 2, [32]). As not all IPD meta-analyses include such assessments or may not describe them explicitly, these are outlined below.

### Were Randomisation, Allocation Concealment, and Blinding Assessed?

Checking that the methods of randomisation and allocation concealment are appropriate is vital in ensuring that a trial provides a fair comparison of interventions (Table 2 [28]). The IPD can be used, for example, to check whether participant characteristics are balanced by arm, as would be expected, or to examine how participants were allocated to intervention arms (Table 2). For example, in a trial in multiple myeloma, similar numbers of patients were allocated to each arm until 1985, when, for a period of about six months, all patients were allocated to the chemotherapy arm [3]. The investigators explained that this was because the radiotherapy equipment was unavailable. Having IPD, it was possible to exclude this small group of nonrandomised patients, ensuring an unbiased analysis [3]. IPD meta-analyses employing such checks safeguard against the inclusion of both nonrandomised trials and nonrandomised participants.

The contact with trial staff that is intrinsic to collaborative IPD meta-analyses can provide useful clarification of, for example, how participants and other study personnel were blinded, allowing the risk of differential care or outcome assessments to be judged with more certainty (Table 2). However, IPD cannot reduce bias or otherwise alter this aspect of trial conduct.

### Were the IPD Checked to Ensure All Randomised Participants Were Included?

Even with adequate randomisation methods, an unbiased comparison of intervention groups can only be guaranteed if all randomised participants are analysed according to the interventions initially assigned, i.e., using an intention-to-treat approach [33–35]. If patients drop out or are excluded from the analysis of a trial in considerable numbers and/or disproportionately by arm, this can cause attrition bias (Table 2, [36]), particularly if the reasons are related to the intervention and outcome. For example, in 14 IPD cancer meta-analyses incorporating 133 trials, between 0.3% and 38% of randomised patients were excluded from the original analyses of 69% of trials, often with more being excluded from the research arms [37]. Around 1,800 excluded patients were reinstated when IPD were collected, and without them the meta-analyses results would have been biased towards the research treatments, albeit to a small degree in most cases [37]. However, for one meta-analysis in soft tissue sarcoma [38], the evidence for a benefit of chemotherapy on survival was stronger when based only on those patients included in the original trial analyses (hazard ratio = 0.85, 95% CI = 0.72–1.00, *p* = 0.06), compared to when all possible patients were included (hazard ratio = 0.90, 95% CI = 0.77–1.04, *p* = 0.16) [37], which could have affected the clinical interpretation. Thus, to help minimise and potentially eliminate attrition bias, IPD should be checked to ensure that data on all or as many randomised participants as possible are included. If there is good reason to exclude certain participants, then this should be prespecified as part of the meta-analysis eligibility criteria and such exclusions applied consistently across trials, to avoid bias [37]. Taking this approach of making consistent (clinically plausible) exclusions across trials in the sarcoma meta-analysis produced very similar results (hazard ratio = 0.91, CI = 0.78–1.07, *p* = 0.28) to those based on all patients.

### Were All Relevant Outcomes Included?

Outcome reporting bias arises if the effect of an intervention varies by outcome, and only particular outcomes are reported, for example, those showing benefit (Table 2). Such bias can be compounded in systematic reviews of published aggregate data [39], because by definition they can only include reported outcomes. Obtaining additional outcome data, as part of the IPD collection process, can resolve this problem, as demonstrated in a systematic review of laparoscopic versus open surgery for the repair of inguinal hernia. Based on published aggregate data from the three trials that reported it, the risk of persistent pain was found to be significantly greater with laparoscopic repair (odds ratio = 2.03, 95% CI = 1.03–4.01) [40]. Yet, when IPD from a further 17 trials (that did not publish results for this outcome) were added, the combined results showed that the risk of persistent pain was actually less with laparoscopic repair (odds ratio = 0.54, 95% CI = 0.46–0.64, *p* < 0.001) [40]. Moreover, the much narrower confidence intervals show that these results are far more reliable. Therefore, to avoid bias and provide a balanced view of benefits and harms, IPD meta-analyses should include data on all available key outcomes, bearing in mind that there may be some that have been collected but not reported in the original trial publication.

### Was the Quality of Time-to-Event Outcome Data Checked?

For time-to-event outcomes, such as survival or time to resolution of symptoms, bias can occur if participants are observed more frequently or for a longer duration on one intervention arm compared to another. This is because it can make it appear that event rates are higher in that arm (Table 2). Even if follow up is similar by arm, trials that stop early or have follow-up that is too short might produce results that are transitory or fail to pick up long-term effects. For example, in an IPD meta-analysis of chemotherapy in addition to local treatment for soft tissue sarcoma [38], median follow up for survival was reported for seven of the included trials and ranged from 16 to 64 months. Investigators supplying IPD were able to extend the median follow up for these trials to between 74 and 204 months [32], which allowed a more reliable examination of the effects of chemotherapy in the longer term [38]. Thus, with IPD, both the pattern and extent of follow up should be checked, and where relevant, additional follow up obtained to rectify any imbalances and provide more up-to-date and reliable outcome data (Table 2). Of course, this may not be practical or necessary if most or all events have already taken place.

## Were the Methods of Analysis Appropriate?

The outcomes and effect measures of interest in an IPD meta-analysis are often the same as those used in a meta-analysis of aggregate data, such as relative risks, odds ratios, or risk differences for dichotomous outcomes; mean differences for continuous outcomes; and hazard ratios for time-to-event outcomes. However, having IPD can allow harmonisation of outcome definitions across trials, analytical assumptions to be checked, and effect measures to be derived directly from the analysis of the collated data (Table 1), with no need to rely on the original trial analyses. Alternative effect measures can be computed and synthesised, if desired. As more complex, nonstandard methods may be used for meta-analyses based on IPD, we outline some key principles to look for, noting that analyses to investigate publication and data availability bias have already been discussed.

### Were the Methods of Assessing the Overall Effects of Interventions Appropriate?

As participants in an IPD meta-analysis are recruited according to different trial protocols, combining IPD across trials, as though it comes from a single “mega” trial, could lead to biased comparisons of interventions and overprecise estimates of effect (Table 2). For example, the effect of nicotine gum on smoking cessation was shown to be severely attenuated and too precise when the analysis treated the IPD as though it belonged to one trial (odds ratio = 1.40, 95% CI = 1.02–1.92) rather than multiple trials (odds ratio = 1.80, 95% CI 1.29–2.52) [41]. Thus, a key component of the analysis is to stratify or account for clustering of participants [41]. To date, two-stage meta-analysis of IPD is the most common way of combining data while preserving participants’ trial membership [42,43]. In the first stage, estimates of effect are derived from the IPD for each trial, and in the second stage, these are combined using methods analogous to most meta-analyses of aggregate data. Thus, standard fixed-effect (e.g., Peto, Mantel-Haenszel, and inverse-variance [44,45]) and random effects methods (e.g., Dersimonian and Laird [46]) can be utilised to derive estimates of the intervention effect, with the latter helping to account for any differences in effect across trials (heterogeneity), and standard statistics (e.g., study weights and I^2^) and forest plots can be produced.

Alternatively, a one-stage model [42] can be used to estimate intervention effects while stratifying or otherwise accounting for differences between trials [47–50]. This is typically a regression model such as linear, logistic, or Cox regression, with either a separate term for each trial or one that varies across trials via a random effect. Based on theoretical considerations [51–53], simulation studies [43,54], and case studies in epilepsy [54], cancer [43], maternal health [55], and childhood infections [56], one-stage and two-stage methods usually produce similar meta-analysis results. For example, the effects of anti-platelets on pre-eclampsia in pregnancy from a two-stage (relative risk = 0.90, 95% CI = 0.83–0.96) and one-stage model (relative risk = 0.90, 95% CI = 0.83–0.97) were almost identical [55]. However, when included studies are small and/or when an effect is large [43,54] or events are rare [57], there can be some bias in a two-stage analysis, because some of the assumptions in the second stage are potentially inappropriate. Thus, for many, the one-stage approach is preferable, although it makes it more difficult to calculate the standard meta-analysis statistics directly, and the complexity can make interpretation of the results less accessible to those who are used to the more familiar two-stage approach. Hence, researchers often will do a one-stage analysis to obtain estimates of effect and use a two-stage analysis to obtain further statistics and forest plots. It is important, therefore, that the choice of one or two-stage analysis is specified in advance or that results for both approaches are reported [55].

### Were the Methods of Assessing If Effects of Interventions Varied by Trial or Participant Characteristics Appropriate?

Exploring whether intervention effects vary or are modified by trial characteristics, such as the duration of the intervention or dose of drugs, and the nature of these interactions is another important aspect of meta-analysis. For both aggregate data and IPD, it is reasonable to investigate these sorts of trial-level interactions using subgroup analyses, whereby intervention effects are compared between groups of trials, or meta-regression in which the change in overall intervention effect in relation to trial characteristics is investigated [58].

A frequent motivation, however, for collecting IPD is the ability to investigate interactions between overall intervention effects and individual participant-level characteristics, such as age or stage of disease or risk of an outcome. This is important for clinical decision-making and policy, in which the aim is to target interventions at those who are most likely to benefit or are least likely to be harmed by them. Single trials are typically underpowered to detect such interactions, and meta-regression of aggregate data is usually inadequate for the purpose, because it also has low power and only permits trial-level interactions between overall intervention effects and summaries of participant characteristics, such as mean age. The latter may not reflect the genuine relationships between intervention effects and individual participant characteristics (Table 2, [59]). Similarly, comparing a two-stage IPD meta-analysis of intervention effects in one participant group with another, for example, effects in men and women, using a test for interaction (sometimes called subgroup analyses) is common in IPD meta-analysis but prone to bias and best avoided [60]. Rather, the focus should be on estimating the individual participant-level interactions, which is usually only possible with IPD. Again, this can be achieved in two stages, by estimating the interactions separately for each trial in the first stage and then pooling these across trials in the second stage, using meta-analysis techniques [60], For example, using this methodology, there was no clear evidence of a relationship between lymph node involvement and the effect of postoperative radiotherapy on survival in non-small cell lung cancer and (interaction hazard ratio = 0.91, 95% CI = 0.74–1.11, *p* = 0.34) [60]. In contrast, using the flawed subgroup analysis approach, the effect of postoperative radiotherapy on survival was erroneously found to vary with the number of lymph nodes affected (chi-square test for trend, df = 1, *p* = 0.016) [61].

Alternatively, a one-stage approach that incorporates an intervention by participant covariate interaction term in a regression model while also accounting for clustering of patients within trials (as described above) can be used. This must carefully separate out this individual participant-level interaction from any trial-level interactions [60,62]. Uniquely, the one-stage approach allows the relative influence of multiple trial and patient characteristics on any intervention effect to be considered simultaneously, which can help deal with confounding [63] and may provide greater clinical insight. When there is no particular evidence of a differential effect by trial or participant characteristic, emphasis should be placed on the overall result.

To allow appropriate interpretation, results of all such analyses should be reported in full and should include effect sizes and confidence intervals for each meta-analysis, measures of statistical heterogeneity, and interactions between intervention effects and trial or participant characteristics [27].

## Discussion

We have provided guidance to help those using the results of IPD meta-analyses to inform policy, practice, or research, and those reviewing grant applications or manuscripts relating to IPD meta-analyses, to assess critically their quality (summarised in Box 1). Expert opinion may be required to navigate the more complicated statistical methods. Given that collecting IPD can be a lengthy and costly endeavour, additionally funders might want to ask researchers to explain what value IPD will bring over aggregate data and whether applicants have established the feasibility of the IPD approach, for example, by contacting trial investigators to see whether they are willing and able to provide data [64]. Realistically, many IPD meta-analyses will not be perfect in all respects, but if they are conducted in the context of a systematic review, include a high-proportion of good-quality data and use appropriate analyses, they will most likely provide reliable assessments of the effects of interventions.

**Box 1. Key Questions to Ask When Appraising an IPD Meta-analysis**

1. Is the IPD meta-analysis part of a systematic review?
  - Does it have a clear research question qualified by explicit eligibility criteria?
  - Does it have a systematic and comprehensive search strategy for identifying trials?
  - Does it have a consistent approach to data collection?
  - Does it assess the “quality” or risk of bias of included trials?
  - Are all the methods prespecified in a protocol?
  - Has the protocol been registered or otherwise made available?
2. Were all eligible trials identified?
  - Were fully published trials identified?
  - Were trials published in the grey literature identified?
  - Were unpublished trials identified?
3. Were IPD obtained for most trials?
  - Were IPD obtained for a large proportion of the eligible trials?
  - Was an assessment of the potential impact of missing trials undertaken?
  - Were the reasons for not obtaining IPD provided?
4. Was the integrity of the IPD checked?
  - Were the data checked for missing, invalid out-of-range, or inconsistent items?
  - Were there any discrepancies with the trial report (if available)?
  - Were any issues queried and, if possible, resolved?
5. Were the analyses prespecified in detail?
  - Were the detailed analysis methods included in a protocol or analysis plan?
  - Were the outcomes and methods for analysing the effects of interventions, quantifying and accounting for heterogeneity, and assessing risk of bias included?
6. Was the risk of bias of included trials assessed?
  - Were the randomisation, allocation concealment, and blinding assessed?
  - Were the IPD checked to ensure all (or most) randomised participants were included?
  - Were all relevant outcomes included?
  - Was the quality of time-to-event-outcome data checked?
7. Were the methods of analysis appropriate?
  - Were the methods of assessing the overall effects of interventions appropriate?
    - Did researchers stratify or account for clustering of participants within trials using either a one- or two-stage approach to meta-analysis?
    - Was the choice of one- or two-stage analysis specified in advance and/or results for both approaches provided?
  - Were the methods of assessing whether effects of interventions varied by trial characteristics appropriate?
    - Did researchers compare treatment effects between subgroups of trials or use meta-regression to assess whether the overall treatment effect varied in relation to trial characteristics?
  - Were the methods of assessing whether effects of interventions vary by participant characteristics appropriate?
    - Did researchers estimate an interaction separately for each trial and combine these across trials in a two-stage fixed effect or random effects meta-analysis? Or;
    - Did researchers incorporate one or more a treatment by participant covariate interaction terms in a regression model, whilst also accounting for clustering of participant, separating out this individual participant-level interaction from any trial-level interactions?
  - If there was no evidence of a differential effect by trial or participant characteristic, was emphasis placed on the overall result?
  - Were exploratory analyses highlighted as such?
8. Does any report of the results adhere to the Preferred Reporting Items for a Systematic review and Meta-analysis of IPD (The PRISMA-IPD Statement) [27]?

With the expected increase in the use of IPD in systematic reviews, as a consequence of the drive for greater sharing of data held within trials, guidance on appropriate methodology will become ever more valuable. Many of the advantages and pitfalls we describe in relation to standard IPD meta-analyses, are transferrable to network IPD meta-analysis of efficacy, and elements of this guidance relating to trial identification, data quality and risk of bias will frequently apply. However, additional research and guidance on appropriate analytical approaches is needed. IPD is also being collected to improve the quality of systematic reviews of diagnostic and prognostic studies [65–67], and to allow necessarily complex, multi-parameter, meta-analysis methods [68] in these reviews. However, such methodology is less well established, and will differ in additional respects. Hence, more specific guidance is needed to facilitate appraisal of these sorts of IPD meta-analyses.

Increasingly, planned methods for IPD meta-analyses can be found in protocols that are registered in the PROSPERO International Prospective Register of Systematic Reviews [69], published or made available by other means. This practice needs to be encouraged, as there is empirical evidence that reporting of search strategies, data availability [12,42] and methods of analysis [42] is variable, and anecdotal evidence that the methods for checking data and risk of bias are frequently absent from IPD meta-analyses publications. Although missing information can be sought from researchers, to enable discerning appraisal, clearly reporting should also be improved. Researchers conducting PD meta-analyses often target high impact print journals [70], and restrictions on the number of words, figures and tables, may have led them to condense or omit particular methods or results. They can now utilise online journals, and the increasing opportunities offered by print journals to publish material online, to alleviate these issues. Journals can help by being responsive to the need for full reporting of IPD meta-analyses. Moreover, those conducting IPD meta-analyses should follow the PRISMA reporting guidelines developed specifically for the IPD approach [27].

Taking steps to identify well designed and conducted IPD meta-analyses of efficacy will help ensure that clinical practice and research is informed by robust evidence about the effect of interventions.

## Abbreviations

AD: aggregate data
IPD: individual participant data
RCT: randomised controlled trial
